# Patterns of service utilization among youth with substance use service need: a cohort study

**DOI:** 10.1186/s13011-023-00572-9

**Published:** 2023-11-03

**Authors:** Nikki Ow, Kirsten Marchand, Guiping Liu, Emilie Mallia, Steve Mathias, Jason Sutherland, Skye Pamela Barbic

**Affiliations:** 1 Department of Occupational Science and Occupational Therapy, Faculty of Medicine, The University of British Columbia, Vancouver, BC V6T 1Z4 Canada; 2 Centre for Advancing Health Outcomes, 570-1081 Burrard Street, Vancouver, BC V6Z 1Y6 Canada; 3 Providence Research, 1081 Burrard Street, Vancouver, BC V6Z 1Y6 Canada; 4 Centre for Health Services and Policy Research, School of Population and Public Health, Faculty of Medicine, University of British Columbia, Vancouver, BC V6T 1Z4 Canada; 5 Foundry Central Office, 201-1190 Hornby Street, Vancouver, BC V6Z 2K5 Canada; 6 Department of Psychiatry, Faculty of Medicine, The University of British Columbia, Vancouver, BC V6T 1Z4 Canada

**Keywords:** Mental health, Substance use, Youth and young adults, Health services

## Abstract

**Background:**

Integrated youth services (IYS) are vital to addressing the needs of youth who use substances. Evidence on the characteristics of youths accessing these services and the types of services accessed have been limited. The objectives were to identify sociodemographic, self-reported health and mental health, patterns of service utilization (service type and frequency of visits) among youths with different levels of substance use service needs (low, moderate, and high), and to estimate the extent to which substance use service needs, self-reported health and mental health influenced the frequency of visits and types of service utilized.

**Methods:**

Data were collected from youth (12–24 years) accessing IYS centres in Canada. Information on socio-demographic factors, substance use in the last month, self-rated health measures, number of service visits, and type of services utilized were included. Poisson regression was used to estimate the relationship between substance use needs and number of service visits and the different type of services utilized.

**Results:**

Of 6181 youths, 48.0% were categorized as low substance use service needs, 30.6% had moderate needs and 21.4% had high needs, with higher proportion of men in the high needs group. Mental health and substance use (MHSU) services were utilized the most across all three groups, followed by counseling. The median number of visits was 4 for the low and moderate needs group and 5 in the high needs group. People with high service needs had 10% higher rate of service visits and utilized 10% more services than people with low service needs (service visits: RR = 1.1 (95%CI: 1.1–1.2); service type: RR = 1.1 (95%CI:1.0-1.1)). The rate of service visits increased by 30 to 50% and the number of services increased by 10–20% for people who rated their health good/fair/poor. Similarly, the rate of service visits increased by 40 to 60% and the number of services increased by 20% for people who rated their mental health good/fair/poor.

**Conclusions and impacts:**

Our study highlighted that regardless of service needs, youth who use alcohol and drugs have complex intersecting needs that present once they access integrated youth services.

**Supplementary Information:**

The online version contains supplementary material available at 10.1186/s13011-023-00572-9.

## Introduction

Substance use in young people is an important public health concern in North America [1, 2]. According to the Global Burden of Disease Survey in 2013, the burden of substance use disorder is substantially higher in North America than other parts of the world [3, 4]. In Canada, substance use is one of the top causes of death in youths aged 19 and below, with an average of 24 deaths per year by drug toxicity [5]. Compared to other age groups, youths aged 15 to 24 years reported the highest rate of substance misuse [6]. Nearly 30% of youths in this age group reported past year cannabis use and this rate is twice that of adults aged 25 to 64. In younger youths aged 15 to 19 [7]. Alcohol was reported as the most common substance among youth, followed by cannabis, and psychoactive pharmaceuticals like stimulants and prescription pain medication [8]. In this age group, approximately 46% reported past year alcohol use, 19.4% reported past year cannabis use [7, 8]. Poly-substance use has also been on the rise among youth in Canada [9].

Adolescent and early adulthood are important periods of physical, emotional, and social development. Evidence from neuroscience has shown that the brain maturation process continues until about 25 years old and cognitive refinement associated with decision making and risk-taking occurs during the period of adolescence, making adolescents particularly vulnerable to substance use [10–12]. Alcohol and drug use during adolescent and early adulthood can significantly impede key developmental milestones. Research has shown that substance use at an early age has been associated with poorer social and health outcomes in later life, such as academic challenges, mental health conditions, involvement with justice system, and many other physical health issues [2, 13–16]. The onset of many other mental health conditions also occurs during this period of development, making it critical for the healthcare system to engage and intervene during this period of adolescence and young adulthood [17, 18].

Youth who use alcohol and drugs experience multiple intersecting health and social challenges and therefore require comprehensive interventions that adapt to their evolving needs. Hence, accessible, multidisciplinary, multi-faceted and youth-centred healthcare is vital to addressing this issue [19, 20]. Integrated youth services (IYS) have been proposed as a solution to this [21, 22]. The IYS model aims to improve the quality of mental health and substance use services for youth by providing multiple services in youth-specific settings [21, 23]. Internationally, there is a range of service models used but there are similar underlying principles such as improving access to health care, early intervention, youth friendly settings, using an evidence- informed approach, and youth and family engagement in the provision of care [22, 24, 25]. IYS networks also commonly have collaboration and partnerships with various agencies and stakeholders to help provide multidisciplinary care like social, educational, employment services, housing support, and income assistance [24]. Examples of IYS organizations in Canada include Foundry in British Columbia (BC), Access Open Minds, a pan-Canadian youth mental health network, and the Youth Wellness Hubs in Ontario [26–28]. While there is some evidence showing that IYS networks have been successful in improving accessibility of healthcare to youths, evidence on the characteristics of youths accessing these services in Canada, the types of services accessed, and the effectiveness of IYS models have been limited [23, 27, 29]. Most studies on IYS models in Canada were descriptive and did not provide evidence for the effectiveness of IYS [26, 27]. However, there is some evidence indicating that youth made more improvements in recovery and health outcomes with more treatment visits however, this was not stratified by substance use service needs [23]. In addition, youth who were unlikely to improve with more intervention often have more severe symptoms and situations. Research has shown that youth with more severe mental health issues are more likely to suffer from the consequences of it, affecting areas like education, employment, social isolation and poorer physical health [30, 31], making it likely that people with higher substance use service needs may have different social demographics and health outcomes [23, 31]. Emerging models of mental health and substance use care for youth involve the matching of interventions to the individual’s clinical and social profile, hence understanding individual characteristics will allow for personalization of care options and preventive care efforts in youth substance use in Canada [22, 32]. Knowledge of substance use service needs in youth can provide insight into the potential burden of disease in later adulthood. This will also help in identifying potential service gaps in health and social services to improve accessibility to healthcare for youth who use substances.

### Objectives

Hence, the objectives of this study were:

1. To identify patterns of service utilization among youths aged 12 to 24 years in a Canadian IYS setting.
2. To identify the demographic and self-reported health and mental health of youths with different levels of substance use service needs (low, moderate, and high) in an IYS setting, and,
3. To estimate the extent to which substance use service needs, self-reported health (SRH) and mental health (SRMH) influenced the frequency of visits and type of service utilized.

We hypothesized that youth with low substance use service needs would have less service visits and utilized fewer service types. We also hypothesize that youth with poorer self-reported health/mental health outcomes will have more service visits and utilize more services.

## Methods

### Study design and setting

This was a retrospective cohort study using data from youth (ages 12–24) accessing IYS across Foundry British Columbia’s 11 community-based centres between May 1, 2018, and January 31, 2022. As of May 2023, Foundry is a network of 15 community IYS centres and a virtual care service across BC [27]. Foundry centres integrate five core service streams: physical and sexual health, mental health, substance use, and social and peer support services. Foundry’s services are delivered by a team of interdisciplinary healthcare professionals (physicians, nurses, counselors, and peer support specialists). Foundry routinely collects demographic, self-reported health measures, and clinical service data among youths accessing Foundry services. Self-reported socio-demographic data were collected at one time point only, at the initial visit to any of Foundry’s centres and online service. Depending on the service utilized, data can be collected in person or online. All centres reported service count and type data which was collected at end of every service visit. These data are collected for research and evaluation purposes, with the broader goal of informing Foundry’s service design and delivery and systematically studying the profile and outcomes of youth accessing IYS services in one of Canada’s largest networks of IYS centres. The dataset includes information on socio-demographic factors, alcohol and substance use in the last month, self-rated health measures, and services utilized. This study has been approved by the Research Ethics Board at Providence Health Care/University of British Columbia (H22–00522). This dataset has been previously described and analyzed in a previous study by our team [33].

### Variables

For this study, we analyzed all data on demographics and health measures and end of visit (EOV) forms to identify the health and service utilization patterns. Social demographics and health measures were self-reported and collected at youth’s first visit to Foundry.

The EOV forms were completed by the service provider at the end of each service visit and were used to identify the method of service delivery (i.e., in-person, virtual, outreach) and the type of service(s) offered at each visit. The EOV forms provides information on which services that clients utilized. Each youth can have multiple EOV forms over time and the total number of EOV forms each youth had reflected the total number of visits. Each EOV form can reflect multiple service types, the types of services utilized on the youth’s EOV form(s) reflected the type of services utilized. The sample analyzed includes everyone who has at least one complete EOV form.

#### Exposure variables

##### The Global appraisal of individual needs – short screener (GAIN-SS) substance problem scale (SPS) [34]

The GAIN-SS is a validated screening tool, developed from a diverse sample of youths aged 10 to 17, aimed to identify those who would have a behavioral health disorder in four dimensions (internalizing disorders, externalizing disorders, substance disorders, and crime/violence) [34]. For this study, only the results on the SPS were used as the objective of the study was to identify patterns of service utilization in youth with substance use needs. The SPS consists of five items. Responses are given according to how recent the problem is: 3 = past month; 2 = 2 to 12 months ago; 1 = 1 + years ago; 0 = never. A total score is obtained through counting the number of symptoms endorsed in the past month on the substance disorder subscale (i.e. total number of 3s and 2s).

Respondents can be classified into three groups reflecting their substance use service needs based on the score:

- Low: score of 0; low needs.
- Moderate: score of 1 to 2; moderate needs.
- High: score of 3 or more; high needs.

The following self-reported health measures were also analyzed in this study.

1. General health perception was measured using the single item self-rated health (SRH) (“in general, how would you say your health is”) from the RAND-36 [35]. Categorical response options include excellent, very good, good, fair, and poor.
2. General perception of mental health was measured using the single item self-rated mental health (SRMH) (“in general, how would you say your mental health is”). Categorical response options include excellent, very good, good, fair, and poor.

#### Outcome variables

Two variables were of interest for identifying service utilization patterns in this IYS setting where youth might engage with multiple service types (from the five core service streams) on a given date: (1) a ‘visit’ was defined and counted when an EOV form had a unique service type selected; (2) ‘Service type’ was defined and counted by what service was selected by the service provider in the EOV form. The following service types were documented in the EOV form:

1. Sexual health – includes contraception prescription and sexual health counseling.
2. Physical health – primary care health services.
3. Mental health and substance use (MHSU) – Access to psychiatrist, psychologist, nurse, counselor, and peer support worker, tailored treatment for each individual.
4. Walk-in counseling – clinical counseling.
5. Navigation – guidance of Foundry’s services with a peer support worker.
6. Youth peer support – peer support through phone or online.

Further information on Foundry services can be found at https://foundrybc.ca/.

For the main analysis, the outcome variables were the total number of service visits over the whole study period (2018 to 2022) and the number of different service types utilized (1–6).

### Analysis

Data from all youth who completed the GAIN-SS were analyzed. Distributional parameters were used to summarize demographic, health, and social characteristics of youths with different levels of substance use service needs. Descriptive statistics were used to characterize the number of visits and service types per youth. To interpret the magnitude of difference between groups, important differences were demonstrated by a difference of more than 10% for categorical variables and more than ½ standard deviation for continuous variables [36, 37]. Due to the fewer cases of people reporting excellent health and mental health, we combined the excellent and very good categories in SRH/SRMH. Ratings of SRH and SRMH were compared to recent Canadian results of SRH and SRMH of youth aged 12 to 17 years in Vancouver [38].

#### Bias

Raw data on the demographic characteristics were provided, only variables that showed important differences between groups (age and gender) were analyzed and adjusted for in all models. Interaction between the variables were tested. Sensitivity analyses included univariate analysis of all variables. To account for differences because of missing data, comparison of responders and non-responders of the GAIN-SS were presented.

#### Statistical methods

To estimate the extent to which substance use service needs, health measures influence the frequency of visits and the number of types of services utilized, multivariable Poisson regression using generalized linear models were used as our data violated the proportional odds assumption for ordinal regression. In multivariate models, substance use levels, self-rated health and self-rated mental health were included. Inclusion of the health outcomes were driven by the clinical reasoning and literature review as mentioned above. Regression (β) coefficients, standard errors (S.E), rate ratios (RR) and 95% CI were presented. Analysis was conducted using the PROC GENMOD function from SAS® version 9.4. Of note, in SAS, a scaling parameter was called to control the impacts of over-dispersion in the data [39].

## Results

Characteristics of the study sample are presented in Table 1. A total of 6181 unique youths had at least one end of visit (EOV) record. Based on the results of the GAIN-SS, 48.0% were categorized as having low substance use needs, 30.6% had moderate substance use needs and 21.4% had high needs. Approximately 60% of the sample identified as female, 49.2% were between 19 and 24 years of age and 63.7% were white. A higher proportion of the high needs group were male, compared to the low needs group (41.7% vs. 28.5%). Youths in both the moderate and high substance use service needs groups were older, with the majority aged 19 to 24 years (moderate: 57.6%; high: 53.3%). More than half of the youth (55.7%) in the lower needs group did not have any existing health condition compared to 40.1% in the high needs group. There were a higher proportion of people who were not in education or employed in the high service needs group (high: 21.4% vs. low: 12.0%). Across all groups, more than 80% were in secured housing.

**Table 1:** Descriptive characteristics of sample

	Low needs (N = 2964)	Moderate needs (N = 1897)	High needs (N = 1320)	Total (N = 6181)
**Gender, n (%)**				
Women	1789 (60.4)	1072 (56.5)	683 (51.7)	3544 (57.3)
Men	**844 (28.5)**	635 (33.5)	**550 (41.7)**	2029 (32.8)
Diverse/other	331 (11.2)	190 (10.0)	87 (6.6)	608 (9.8)
**Age, n (%)**				
12–14	497 (16.8)	121 (6.4)	100 (7.6)	718 (11.6)
15–18	1196 (40.4)	665 (35.1)	501 (38.0)	2362 (38.2)
19–24	**1243 (41.9)**	**1093 (57.6)**	**703 (53.3)**	3039 (49.2)
**Race, n (%)**				
White	1888 (63.7)	1227 (64.7)	821 (62.2)	3936 (63.7)
non-White	1014 (34.2)	639 (33.7)	474 (35.9)	2127 (34.4)
**Diagnosed health conditions**				
None	**1651 (55.7)**	905 (47.7)	**529 (40.1)**	3085 (49.9)
Fetal Alcohol Syndrome	9 (0.3)	2 (0.1)	13 (1.0)	24 (0.4)
Learning disabilities	127 (4.3)	72 (3.8)	42 (3.)	241 (3.9)
Brain injury	49 (1.7)	28 (1.5)	24 (1.8)	101 (1.6)
ADD or ADHD	343 (11.6)	322 (17.0)	221 (16.7)	886 (14.3)
Cognitive problems	42 (1.4)	28 (1.5)	31 (2.3)	101 (1.6)
≥2 health conditions	375 (12.7)	199 (16.4)	269 (20.4)	956 (15.5)
**Education, n (%)**				
Elementary	486 (16.4)	111 (5.9)	104 (7.9)	701 (11.3)
Some high school	1078 (36.4)	669 (35.3)	497 (37.7)	2244 (36.3)
High school	884 (29.8)	757 (39.9)	483 (36.6)	2124 (34.4)
Certificate	208 (7.0)	187 (9.9)	120 (9.1)	515 (8.3)
University	127 (4.3)	86 (4.5)	40 (3.0)	253 (4.1)
**In education or employment, n (%)**				
Yes	2420 (81.6)	1515 (79.9)	958 (72.6)	4893 (79.2)
No	357 (12.0)	282 (14.9)	282 (21.4)	921 (14.9)
**Housing, n (%)**				
Secure	2615 (88.2)	1679 (88.5)	1118 (84.7)	5412 (87.6)
Group home	33 (1.1)	23 (1.2)	14 (1.1)	70 (1.1)
Insecure	111 (3.7)	73 (3.8)	95 (7.2)	279 (4.5)
Other	65 (2.2)	48 (2.5)	31 (2.3)	144 (2.3)

Important differences (> 10% or > 1/2SD) are highlighted in bold

* Low needs (score of 0 on the SPS subscale); moderate needs (score of 1–2 ), High needs (score of 3 and above)

†Diverse/other genders include non-binary, trans female, trans male, agender, two-spirit and unsure/questioning

Table 2 shows self-reported substance use in the past month. In the whole sample, about 25% of youth identified as regular users of cannabis (28.9) and tobacco (27.3%). Youths with low substance use needs reported irregular use of alcohol (60.0%), and many have not tried cannabis (39.3%) and alcohol (46.1%), while youths with moderate substance use service needs reported irregular use of alcohol (77.3%) and regular user of cannabis (44.3%). Youths with high needs reported irregular use of alcohol (62.3%) and regular use of cannabis (59.6%) and tobacco (56.7%). Compared to youths with low (2.2%) and moderate substance use service needs (12.4%), 37.4% youths with high needs reported illicit drug use in the past month.

**Table 2:** Substance use in the past month

	Low needs (N = 2964)	Moderate needs (N = 1897)	High needs (N = 1320)	Total (N = 6181)
**Past month**				
**Alcohol, n (%)**				
Never tried	**633 (21.4)**	**27 (1.4)**	**9 (0.7)**	669 (10.8)
Irregular	**1778 (60.0)**	**1466 (77.3)**	823 (62.3)	4067 (65.8)
Regular	**24 (0.8)**	**145 (7.6)**	**309 (23.4)**	478 (7.7)
Missing	529 (17.8)	259 (13.7)	179 (13.6)	967 (15.6)
**Cannabis, n (%)**				
Never tried	**1164 (39.3)**	**111 (5.9)**	**24 (1.8)**	1299 (21.0)
Irregular	**1033 (34.9)**	**657 (34.6)**	**276 (20.9)**	1966 (31.8)
Regular	**160 (5.4)**	**840 (44.3)**	**787 (59.6)**	1787 (28.9)
Missing	607 (20.5)	289 (15.2)	233 (17.7)	1129 (18.3)
**Tobacco, n (%)**				
Never tried	**1365 (46.1)**	**305 (16.1)**	**64 (4.8)**	1734 (28.1)
Irregular	746 (25.2)	607 (32.0)	318 (24.1)	1671 (27.0)
Regular	**314 (10.6)**	**627 (33.1)**	**748 (56.7)**	1689 (27.3)
Missing	539 (18.2)	358 (18.9)	190 (14.4)	1087 (17.6)
Illicit drug use*, n (%)				
Yes	**64 (2.2)**	**235 (12.4)**	**491 (37.2)**	790 (12.8)
Not reported	2900 (97.8)	1662 (87.6)	829 (62.8)	5391 (87.2)

Important differences (> 10% or > 1/2SD) are highlighted in bold

* Low needs (score of 0 on the SPS subscale); moderate needs (score of 1–2 ), High needs (score of 3 and above)

†Illicit drug use refers to cocaine, heroin/fentanyl, hallucinogens, MDMA & amphetamine

Results of both self-rated health and self-rated mental health were reported in Table 3.

**Table 3:** Self-rated health (SRH) and self-rated mental health (SRMH) compared to Canadian norm

	A: Low needs (N = 2968)	B: Moderate needs (N = 1900)	C: High needs (N = 1321)	Total (N = 6189)	Norms for youth aged 12 to 17 years [38]
**SRH, n (%)**					
Excellent/very good	502 (16.9)	219 (11.5)	105 (8.0)	826 (13.4)	77%
Good	**1165 (39.3)**	698 (36.8)	**373 (28.3)**	2236 (36.2)	
Fair	**862 (29.1)**	656 (34.6)	**531 (40.2)**	2049 (33.1)	4.4%^†^
Poor	**212 (7.2)**	204 (10.8)	**227 (17.2)**	643 (10.4)	
**SRMH, n (%)**					
Excellent/very good	115 (3.9)	59 (3.1)	16 (1.2)	190 (3.1)	77%
Good	436 (14.7)	176 (9.3)	89 (6.7)	701 (11.3)	
Fair	1187 (40.0)	730 (38.5)	446 (33.8)	2363 (38.2)	7.0%^†^
Poor	**991 (33.4)**	805 (42.)	**673 (51.0)**	2469 (39.9)	

Important differences (> 10% or > 1/2SD) are highlighted in bold

* Low needs (score of 0 on the SPS subscale); moderate needs (score of 1–2 ), High needs (score of 3 and above)

†Proportion of people who had a fair/poor rating of SRH/SRMH

For self-rated health, only 10.4% of the sample rated their health poor but 39.9% rated their mental health as poor. A total of 56.3% and 48.3% of youths in low and moderate service needs rated their health good, very good and excellent compared to 36.3% of youths with high service needs. This value was more than 10% difference (36.3% vs. 56.3/48.3%). Approximately 17% of youths in the high needs group rated their health as poor compared to 7.2% in the low service needs group. For mental health, 51% of youths in the high service needs rated their mental health as poor, compared to 33.4% and 42.4% of youths in low and moderate service needs, respectively. Compared to Canadian norms [40–42], the proportion of youths who rated their health and mental health excellent/very good (13.3% and 3.1% respectively) were much lower than Canadian norms.

The frequency of visits across all service types is shown in Table 4. The median number of sessions was 4.0 for the total sample but the median number of visits for people in the high substance needs group was 5.0. More than 80% of youth across all groups utilized two or more services. Mental health and substance use (MHSU) services were utilized the most across all three groups with more than 60% of people utilizing this service. The second most utilized service across all groups was counseling. In terms of service visits, most youths had less than three service visits across all service types but approximately 27% of youths had more than four visits during the study period.

**Table 4:** Type of services utilized and frequency of visits across groups

	Low needs (N = 2964)	Moderate needs (N = 1897)	High needs (N = 1320)	Total (N = 6181)
**Median number of visits**	4.0	4.0	5.0	4.0
**Engaged in one service or more, n (%)**	2456 (82.9%)	1577 (83.1%)	1134 (85.9%)	5167 (83.6%)
**Physical health**^†^, **(range: 1–42), n (%)**				
1–3	599 (20.2)	371 (19.6)	272 (20.6)	1242 (20.1)
≥4	505 (17.0)	307 (16.2)	208 (15.8)	1020 (16.5)
**Sexual health (range: 1–19), n (%)**				
1–3	391 (13.2)	249 (13.1)	188 (14.2)	828 (13.4)
≥4	210 (7.1)	160 (8.4)	105 (8.0)	475 (7.7)
**Any MHSU services (1–51), n (%)**				
1–3	1230 (41.5)	757 (39.9)	522 (39.5)	2509 (40.6)
≥4	606 (20.4)	433 (22.8)	341 (25.8)	1380 (22.3)
**Walk-in counselling (range: 1–32), n (%)**				
1–3	880 (29.7)	567 (29.9)	428 (32.4)	1875 (30.3)
≥4	820 (27.7)	519 (27.4)	358 (27.1)	1697 (27.5)
**Navigation**‡ **(range: 1–6), n (%)**				
≥1	229 (7.7)	157 (8.3)	107 (8.1)	493 (8.0)
**Peer support (range: 1–94), n (%)**				
≥1	112 (3.8)	81 (4.3)	59 (4.5)	252 (4.1)
**Mode of access, n (%)**				
In Person	2875 (96.9)	1849 (97.3)	1291 (97.7)	6015 (97.2)
Outreach	26 (0.9)	8 (0.4)	16 (1.2)	50 (0.8)
Virtual Care	67 (2.3)	43 (2.3)	14 (1.1)	124 (0.02)

Important differences (> 10% or > 1/2SD) are highlighted in bold

** Low needs (score of 0 on the SPS subscale); moderate needs (score of 1–2 ), High needs (score of 3 and above)

†Physical health = primary care

‡Specific to some centers

Table 5a and 5b shows results from the Poisson regression analysis for the association between substance use service needs and the frequency of visits and number of service types, respectively. Results of univariate analyses showed that there was no difference on rate of service visits and types of services between the low and moderate substance use service needs groups. People with high substance use service need had 1.2 times (CI: 1.1–12) the service visit rate compared to the people with low substance use service need. All other variables held constant, people with high substance use service needs had 1.1 times (beta coefficients (β) = 0.13, S.E = 0.04) higher service visit rate than people with low substance use service needs. For all other variables held constant, people who identified as men had 0.8 times (β=-0.20, S.E = 0.04) the service visit rate of women while people who identified as other diverse genders had 1.6 times (β = 0.49, S.E = 0.05) the service visit rate of women. For people who identified as other diverse genders, the service utilization rate increased to 1.9 times if they were in the high substance use services needs groups (β = 0.62, S.E = 0.05). In addition, compared to people who rated their health excellent/very good, the rate of service visits increased by 1.3 to 1.5 times for all other ratings (multivariate analysis: good: β = 0.25, S.E = 0.06; fair: β = 0.35, S.E = 0.06; poor: β = 0.39, S.E = 0.08). Similarly, univariate analysis of SRMH showed that compared to people who rated their mental health excellent or very good, all other ratings had 1.8 times (CI: 1.4–2.4) the service utilization rate. Results of multivariate analysis showed that, all other variables held constant, people who rated mental health other than excellent or very good, had 1.4 to 1.6 times the service visit rate of people who rated their mental health excellent/very good (good: β = 0.49, S.E = 0.14; fair: β = 0.43, S.E = 0.14; poor: β = 0.14, S.E = 0.08). For people who rated their health and mental health poor, the service utilization rate increased to 2.1 times that of people who rated their health and mental health excellent or very good (β = 0.75, RR = 2.11). All interaction terms between variables in the multivariate model were not statistically significant.

**Table 5a:** Association between substance use needs, SRH and SRMH on frequency of service visits

Variables		Univariate RR (95% CI)	Multivariate β	S.E	RR (95% CI)
Substance needs	Low		ref		
	Moderate	1.0 (0.9–1.1)	-0.04	0.04	1.0 (0.9–1.0)
	High	1.2 (1.1–1.3)	0.13	0.04	1.1 (1.1–1.2)
Gender	Female		ref		
	Male		-0.20	0.04	0.8 (0.8–0.9)
	Diverse/other		0.49	0.05	1.6 (1.5–1.8)
Self-rated health	Excellent/very good		ref		
	Good	1.3 (1.2–1.5)	0.25	0.06	1.3 (1.1–1.5)
	Fair	1.5 (1.3–1.7)	0.35	0.06	1.4 (1.3–1.6)
	Poor	1.5 (1.3 to 1.8)	0.39	0.08	1.5 (1.3–1.7)
Self-rated mental health	Excellent/very good		ref		
	Good	1.8 (1.4–2.4)	0.49	0.14	1.6 (1.2–2.2)
	Fair	1.8 (1.4–2.4)	0.43	0.14	1.5 (1.2–2.0)
	Poor	1.8 (1.4–2.4)	0.36	0.14	1.4 (1.0–1.9)

**Table 5b:** Association between substance use needs, SRH and SRMH on the number of types of services

Variables		Univariate RR (95% CI)	Multivariate β	S.E	RR (95% CI)
Substance needs	Low		ref		
	Moderate	1.0 (1.0–1.0)	0.00	0.02	1.0 (1.0–1.0)
	High	1.1 (1.0–1.1)	0.05	0.02	1.1 (1.0–1.1)
Gender	Female		ref		
	Male		-0.11	0.02	0.9 (0.9–0.9)
	Diverse		0.18	0.02	1.2 (1.1–1.2)
Self-rated health	Excellent/very good		ref		
	Good	1.1 (1.1–1.2)	0.07	0.02	1.1 (1.1–1.2)
	Fair	1.1 (1.1–1.2)	0.10	0.03	1.1 (1.1–1.2)
	Poor	1.2 (1.1–1.3)	0.15	0.03	1.2 (1.1–1.3)
Self-rated mental health	Excellent/very good		ref		
	Good	1.2 (1.1–1.3)	0.13	0.05	1.1 (1.1–1.3)
	Fair	1.2 (1.1–1.3)	0.12	0.05	1.1 (1.1–1.2)
	Poor	1.2 (1.1–1.3)	0.12	0.05	1.1 (1.1–1.2)

In Table 5b, all other variables held constant, compared to people with low substance use service needs, people with high substance use service needs utilized 1.1 times the number of services (β = 0.05, S.E = 0.02). This was the same for both univariate and multivariate analysis. For gender, all other variables held constant, compared to females, males utilized 0.9 times (β=-0.11, S.E = 0.02) the number of services while people who identified as other diverse genders utilized 1.2 times (β = 0.18, S.E = 0.02) the number of services. The number of services utilized increased by 1.3 times for people who are in the high substance use services needs groups who identified with other diverse genders (β = 0.23, RR = 1.26). For the rating of health, all other variables held constant, compared to people who rated their health excellent/very good, the number of services increased by 1.1 to 1.2 times for all other ratings (good: β = 0.07, S.E = 0.02; fair: β = 0.10, S.E = 0.03; poor: β = 0.15, S.E = 0.03). Similarly for mental health rating, the number of services increased 1.2 times for people who did not rate their health excellent or very good (good: β = 0.13, S.E = 0.05; fair: β = 0.12, S.E = 0.05; poor: β = 0.12, S.E = 0.05). For people who rated their health and mental health poor, the number of services increased by 1.3 times that of people who rated their health and mental health excellent or very good (β = 0.27, RR = 1.30). All interaction terms between variables in the multivariate model were not statistically significant.

As a supplementary analysis, the demographics of people who responded to the GAIN-SS SPS were compared to non-responders (supplementary Table 1). Analysis of missing data showed that there were 11.6% of youth aged between 12 and 14 who responded on the GAIN-SS substance use subscale compared to 25.1% of the same age group who did not respond. There were also more people who were in the older age group (19 to 24 years) who responded to the GAIN-SS substance use subscale (responders: 49.2% vs. non-responders: 32.7%).

## Discussion

The objectives of this study were to identify socio-demographic, health characteristics and patterns of service utilization of youth with substance use service needs who accessed IYS services in British Columbia, Canada. Results from analysis showed that in the higher needs group, there was a higher proportion of men, mostly White, a higher proportion were not in school or unemployment and more reported use of illicit drugs. Across the groups, a higher proportion of people with high substance use service needs rated their health or mental health as poor or fair.

Being in the group with higher substance use service needs was associated with increased service visits and increased utilization of different types of services. However, the relative rate for both outcomes was small. This small difference between groups is likely due to the nature of our sample. Research has shown that youths who seek services, including those who identify as being early in their health seeking trajectory, identified multiple service needs and require a wide variety of services regardless of their substance use service needs [43, 44]. In addition, the median number of visits was 4.0, suggesting that continuity of treatment might be an issue. This could be due to the social and structural stigma associated with substance use, hence there is a need to ensure that there is adequate follow up and support for youth with substance use issues [45]. Nevertheless, more than 80% of youths utilized more than two services, suggesting that youths experiencing substance use challenges feel comfortable accessing integrated youth services and require multiple services and supports.

A SRMH and SRH rating of good/fair/poor was independently associated with an increased rate of service visits and increased number of services. This is not surprising as population health studies have shown that an excellent or very good rating of SRH and SRMH is associated with good health habits and higher levels of social support, particularly in youths [40, 46–48]. In older adults with substance use disorders, a poor rating of SRH was associated with increased levels of alcohol and prescription drug use [49]. SRH has also been strongly associated with poor social circumstances and poor well-being especially in people who are marginalized [50]. Similarly, a poor rating of SRMH has been associated with poorer levels of community belonging in people with mental health and substance use disorders [48]. Of note, the relationship between these measures and substance use is not unidirectional, that is, people with poor rating of SRH and SRMH could also have a higher risk of using more substances. Thus, poor ratings of SRMH and SRH may be useful indicators of an increased need for health and social services. These measures are simple one-item measures that can be incorporated in routine substance use care for youth with MHSU disorders [51]. People who report fair/poor SRH and SRMH should be flagged and followed up. Our results showed that this meant that they require more services and follow up.

The patterns of substance use were different across all three groups. In the low service need group, about 40 to 46% of youth have never tried tobacco or cannabis, and only 2% reported illicit drug use. This, compared to the youth with high service needs, where nearly 60% of youth are regular users of cannabis and nearly 40% reported illicit drug use. Interestingly, alcohol has been reported as the most common substance among youth in Canada but only 8% of this cohort were regular users of alcohol [8]. It is possible that because alcohol consumption is deemed more socially acceptable and therefore less serious compared to a drug addiction, hence most people tend not to seek services for alcohol addiction [52, 53].

Demographics of the youth in our sample is comparable to previous research on substance use in community samples of Canadian youth [54]. There was a higher proportion of men in the high service needs group. Results also showed that compared to youth who identified as women, males had lower service utilization rate and utilized lesser number of services. This result is similar to other research which showed that females were more likely than males to use more MHSU services [55–58]. One explanation to this could be that males are more likely to not turn up for treatment, hence fewer service visits, and more unlikely to use more services. Research of community samples of youths with substance use in the United States have found that male adolescents were more likely to follow an escalating course of substance use compared to females, thereby increasing their substance use service needs [59, 60]. These differences may relate to the experiences of substance use among men and women. Our results also showed that gender diverse youths had a higher rate of service visits and utilized more services. This is consistent with current research where higher rates of mental health disorders and higher levels of substance use have been consistently reported among gender diverse youths and young adults [61]. Non-binary gender youths and young adults are particularly vulnerable and more likely to be exposed to discrimination and stigma from a young age [61]. Our results implied that certain genders are going to be at a higher risk of developing a more serious substance use problem. Knowledge of these differences can aid in the planning of prevention strategies, for example, having additional follow up in place for men.

In Canada, mental health and substance use disorders are the largest absolute cause of disability and morbidity in adults especially men, and this pattern typically starts in early adolescence [4]. Access to and efficient delivery of mental health and substance use services for youths is crucial in reducing this burden. Our study offers some insight on the service patterns of help-seeking youth in Canada. While initial access to services might not be an issue to the participants in our study, continued access may have been a problem. Our results suggest that continued engagement with youths is necessary to ensure that youths with substance use service needs continue with treatment.

In addition, identification of demographical differences and less than ideal SRH/SRMH can aid in early identification of people with higher risks and effective tailoring of treatment [62]. Preventive efforts should be applied and incorporated across different settings like school, healthcare system and the community [25, 63, 64]. Additional research is needed to measure the needs of youths with substance use disorders, especially those from diverse backgrounds and to understand the experience of IYS from the perspectives of youths with different substance use service needs.

### Limitations

This study has a few limitations. Firstly, this was a non-random sample of youth who received treatment at Foundry BC. Although data came from all Foundry’s centers across BC, it might be difficult to generalize findings to youth who did not access Foundry’s services. This could have also affected the demographic characteristics of our sample. Based on descriptive statistics, there were no differences between groups on common confounders like housing status, education status, and employment, hence these were not included in the regression analysis.

Further, the GAIN-SS was used to categorize the substance use service needs in this dataset. GAIN-SS is a screening tool that is used to flag potential MHSU disorders and requires a second assessment for confirmation of diagnosis [34]. With regards to the psychometric properties of the GAIN-SS, research has shown that GAIN-SS has low specificity which implies that the probability of detecting true negatives, in this case youth with no substance use service need or very low levels of substance use, is low [65]. While it might be possible that some youths might have been misclassified due to the specificity issues, this issue of specificity is primarily applicable to the whole scale and not the SPS subscale. Comparison of the demographics of responders and non-responders on the GAIN-SS SPS did show that more than half of the non-responders were below 18 years. It might be possible that some items on the GAIN-SS SPS might be difficult for younger adolescents to understand.

Description of the service provided was also dependent on the service provider who fills up the EOV form so there could have been a problem with the categorization of service type. This may have affected the type of services utilized. It would have been ideal to use different measures, but this was not available with this dataset. This highlights the importance using appropriate measurement tools in IYS practice that are fit for purpose to measure the needs of youth and screen them into appropriate services where and when needed.

There was also significant missing data (> 10%) on variables such as diagnosis and substance use in this dataset which appears to be missing not at random (MNAR). This could have affected our socio-demographic results across the different needs group.

Lastly, data from the health measures and GAIN-SS were captured only at baseline which also may not be a true baseline as people may have received MHSU services prior to Foundry. There were no repeated measures of these outcomes, hence, it was not possible to ascertain where the youths are on the recovery trajectory. Service utilization patterns may differ if we had a clearer picture of their recovery trajectory.

## Conclusion

This study offers a unique perspective across 11 communities in British Columbia, Canada. Results showed that the needs of youth who use substances are complex and there is need to improve the quality of interventions provided for youth in Canada [33, 64]. Youth with substance use challenges who are accessing services should be engaged regularly and monitored. Service providers need to measure often and measure better [62]. In an IYS system where different types of services are provided, outcome measurement is also essential for resource allocation. The opportunity exists for IYS models to be developed in different communities across British Columbia and for communities to co-design assessment and intervention processes that are developmentally and culturally relevant to the communities in which the services are being delivered [25, 66].

### Electronic supplementary material

Below is the link to the electronic supplementary material.

Supplementary Material 1

## Data Availability

Data for this study are not publicly available as participants of this study did not agree for their data to be shared publicly.

## Abbreviations

IYS: Integrated youth services
BC: British Columbia
EOV: End of visit form
GAINS-SS: The Global Appraisal of Individual Needs – Short Screener
SPS: Substance Problem Scale
SRH: Self-rated health
SRMH: Self-rated mental health
MHSU: Mental health and substance use
