# Supplementary material for: Patterns of service utilization among youth with substance use service need: a cohort study
Source: Subst Abuse Treat Prev Policy. 2023 Nov 3;18:62. doi: 10.1186/s13011-023-00572-9 (PMC10623844; doi:10.1186/s13011-023-00572-9)
Supplement: Supplementary file 1 — Supplementary Material 1 [file 13011_2023_572_MOESM1_ESM.docx]

**Supplementary Table 1: Comparison of responders and non-responders on the GAIN-SS substance use scale.**

|  | Responders (N=6181) | Non-responders (N=2097) |  |
| --- | --- | --- | --- |
| 1. Gender, n (%) |  |  |  |
| Female | 3544 (57.3) | 1171 (55.8) |  |
| Male | 2029 (32.8) | 631 (30.1) |  |
| Diverse/other | 608 (9.8) | 295 (14.1) |  |
| 1. Age, n (%) |  |  |  |
| 12-14 | **718 (11.6)** | **526 (25.1)** |  |
| 15-18 | 2362 (38.2) | 829 (39.5) |  |
| 19-24 | **3039 (49.2)** | **686 (32.7)** |  |
| Missing | 62 (1.0) | 56 (2.7) |  |
| 1. Ethnicity, n (%) |  |  |  |
| White | 3936 (63.7) | 1215 (57.9) |  |
| non-White | 2127 (34.4) | 790 (37.7) |  |
| Missing | 118 (1.9) | 92 (4.4) |  |
| 1. Highest level of education, n (%) |  |  |  |
| Elementary | 701 (11.3) | 465 (22.2) |  |
| Some high school | 2244 (36.3) | 681 (32.5) |  |
| High school | 2124 (34.4) | 400 (19.1) |  |
| Certificate | 515 (8.3) | 92 (4.4) |  |
| University | 253 (4.1) | 30 (1.4) |  |
| Missing | 344 (5.6) | 429 (20.5) |  |
| 1. In education and/or employment,   n (%) |  |  | |
| No Ed/Emp | 921 (14.9) | 135 (6.4) | |
| Ed/Emp | 4893 (79.2) | 1524 (72.7) | |
| Missing | 367 (5.9) | 438 (20.9) | |
| 1. Current housing type, n (%) |  |  | |
| Secure | 5412 (87.6) | 1719 (82.0) | |
| Group home | 70 (1.1) | 17 (0.8) | |
| Insecure | 279 (4.5) | 26 (1.2) | |
| Other | 144 (2.3) | 35 (1.7) | |
| Missing in blank | 276 (4.5) | 300 (14.3) | |
| 1. Self-rated health, n (%) |  |  | |
| Excellent/very good | 826 (13.4) | 417 (19.9) | |
| Good | 2236 (36.2) | 824 (39.3) | |
| Fair | 2049 (33.1) | 651 (31.0) | |
| Poor | 643 (10.4) | 147 (7.0) | |
| Missing | 427 (6.9) | 58 (2.8) | |
| 1. Self-rated mental health, n (%) |  |  | |
| Excellent/very good | 190 (3.1) | 100 (4.8) | |
| Good | 701 (11.3) | 302 (14.4) | |
| Fair | 2363 (38.2) | 874 (41.7) | |
| Poor | 2469 (39.9) | 745 (35.5) | |
| Missing | 458 (7.4) | 76 (3.6) | |
